# Impact of Integrated Care on Patient-Related Outcomes Among Older People – A Systematic Review

**DOI:** 10.5334/ijic.4632

**Published:** 2019-07-24

**Authors:** Ann E. M. Liljas, Fanny Brattström, Bo Burström, Pär Schön, Janne Agerholm

**Affiliations:** 1 Department of Public Health Sciences, Division of Social Medicine, Karolinska Institutet, Stockholm, SE; 2 Centre for Epidemiology and Community Medicine, Stockholm County Council Health Services, Stockholm, SE; 3 Aging Research Center, Karolinska Institutet and Stockholm University, Stockholm, SE

**Keywords:** integrated care, hospital admission, length of hospital stay, hospital readmission, patient satisfaction, mortality

## Abstract

**Introduction:**

The growing number of older adults with multiple needs increases the pressure to reform existing healthcare systems. Integrated care may be part of such reforms. The aim of this systematic review was to identify important patient-related outcomes of integrated care provided to older adults.

**Methods:**

A systematic search of 5 databases to identify studies comprising older adults assessing hospital admission, length of hospital stay, hospital readmission, patient satisfaction and mortality in integrated care settings. Retrieved literature was analysed employing a narrative synthesis.

**Results:**

Twelve studies were included (2 randomised controlled trials, 7 quasi-experimental design, 2 comparison studies, 1 survey evaluation). Five studies investigated patient satisfaction, 9 hospital admission, 7 length of stay, 3 readmission and 5 mortality. Findings show that integrated care tends to have a positive impact on hospital admission rates, some positive impact on length of stay and possibly also on readmission and patient satisfaction but not on mortality.

**Conclusions:**

Integrated care may reduce hospital admission rates and lengths of hospital stay. However due to lack of robust findings, the effectiveness of integrated care on patient-related outcomes in later life remain largely unknown. Further research is needed to establish the effect of integrated care on these patient-related outcomes.

**Prospero registration number:**

CRD42018110491.

## Introduction

### Ageing populations and the need of changes to care systems

Worldwide, the population of older people is increasing [1]. Older people’s health and social care needs are often complex requiring several health and social care providers and involves multiple transitions between care settings. These care transitions tend to negatively affect the continuity of care including lack of communication between care providers, errors in medication lists and insufficient quality of discharge protocols [3,4,5]. Subsequently, managing the health and social care of older people has become a major challenge [6]. The World Health Organization (WHO) therefore advocates for major reforms to health and social care systems [7] where integration and coordination between health and social care providers is seen as essential to address the needs of the older population [8].

### Integrated care

Integrated care seeks to better coordinate health and social care around the individual’s needs with a commitment to improve the quality of care and overcome fragmented care through ongoing co-productive partnerships [2]. Integrated care can take many different forms. It may, for instance, take place between providers operating at the same level (horizontal integration) (e.g. bringing together acute hospitals) or between providers working at different levels (vertical integration) (e.g. linking hospitals with general practices and community care) [9]. Typologies of integrated care include clinical, organisational and systemic integration. Clinical integration refers to processes within or across professions through use of shared guidelines and protocols, organisational integration refers to co-ordinated provider networks or contracts that bring together separate organisations, and systemic integration involves coherence of rules and policies at all organisational levels. All degrees of integration are concerned with the processes of bringing organisations and professionals together with the purpose of improving the outcomes for patients such as patients’ experience and the quality of care provided [10].

### Health and social care for older people in Sweden

This systematic review is part of a larger research project on integrated care in Stockholm, Sweden, investigating efficient ways of organising health and social care services for older people with complex health problems and severe needs. Sweden ranks high in cross-country comparisons of care for older people [11]. However, in recent years, Sweden has been criticised for inadequate coordination between hospitals, primary care and social care [12]. The Swedish health and social care system is highly decentralised [13]. The healthcare and the social services are managed separately and limited joint work has resulted in inadequate coordination between hospitals, primary care and social care services. Competition and privatisation in the healthcare sector including implementation of patient choice of primary care and social care providers has gradually been introduced in response to criticism for lack of cost control and inefficiency. However, it has turned out that the marketization of the healthcare has also made it more difficult to coordinate the care for those with complex care needs [12].

Since the Community Care Reform in the early 1990s, the number of hospital beds has more than halved and Sweden has now the lowest hospital bed rate per capita in the European Union [14]. In turn, the reduced number of hospital beds has led to shorter average length of stay in hospitals. Since year 2000, a third of the beds in municipal institutional care have been removed and replaced by municipal home help services, which is significantly cheaper [15]. Consequently, an increased number of older people with complex health problems including cognitive impairments are dependent on help to carry out everyday life activities in their homes. However, the reduction in the number of hospital beds and beds in municipal institutional care has not been compensated for by a corresponding increase in home help services. Not only have these structural changes increased the pressure on the municipal home help services but also on primary health care, and resulted in increased numbers of emergency department visits [13]. Therefore, for the Swedish context, hospital admission, length of stay and hospital readmission are outcomes of particular interest to be assessed against integrated care or in relation to it.

### Previous studies on patient-related outcomes of integrated care

Earlier studies assessing patient-related outcomes, typically hospitalisation and lengths of hospital stay, in settings consisting of at least a few components of integrated care, have shown mixed results [16,17,18,19,20,21,22,23,24,25,26,27,28,29]. For instance, some studies have reported positive trends on hospitalisation, lengths of stay and hospital readmission [16,17,18,19,20,22,28]. However, only a few of these studies reported significant improvements in such outcomes [17,19,20]. Similarly, conflicting findings have also been reported in previous studies examining patient-satisfaction [16,17,18,19,20,21,22,23,24,25,26,27,28,30,31,32] and mortality [16,17,19,20,33,34,26,31,29,35], of which some studies have indicated positive trends [16,17,33,34,31,32,35], however effects remain unclear. Further, despite multi-morbidity (having two or more chronic conditions) becoming progressively more common with advanced age [36], this growing body of evidence mainly comes from studies that have evaluated disease-specific interventions. These studies have furthermore used different definitions and various components of integrated care [24,37,38,27]. Reviewing existing research in older adults with multimorbidity using a specific and well-established definition of integrated care has the potential to demonstrate patient-related outcomes of particular importance to integrated care.

### Study rationale

Recent literature has concluded that to increase the effectiveness of integrated care, care of patients with complex needs should be targeted [35]. Thus, a systematic review of existing research on older adults with multimorbidity could help inform future models of integrated care. Further, whilst many previous studies on integrated care have examined a single patient-related outcome such as hospitalisation, fewer studies have considered investigating more than one patient-related outcome [39]. This systematic review focuses on a range of patient-related outcomes including hospital admission, length of hospital stay, hospital readmission, mortality and patient-satisfaction. Hospital admission and length of stay are outcomes of particular importance as they account for the majority of overall healthcare costs and constitute the starting point of further decline in health in older age [40]. Assessing patient-satisfaction is important to understand the care consumers’ satisfaction with the medical services provided and to identify the needs and expectations of the healthcare system [30]. Mortality is of interest to assess as it is one of the strongest indicators of treatment efficacy and considered the hardest outcome criterion conceivable [41].

A systematic review focusing on an array of patient-related outcomes has the potential to become a resource for current work and future research highlighting patient-related outcomes in integrated care settings of particular interest in the ageing population. The outcomes assessed in the current review will further help forming the knowledge base of the extended research project of which this study is part of.

### Purpose

The aim of this systematic review is to investigate a range of patient-related outcomes to identify if any of them may be particularly important when assessing integrated care for older people with multi-morbidity. Patient-related outcomes explored in this review include patient satisfaction, hospital admission, length of hospital stay, hospital readmission and mortality.

### Conceptual framework

A challenge when studying integrated care is the lack of a universal definition of the concept and the many terms used in the literature [42]. To make sure that the studies included in the review were as comparable as possible, we have to the largest extent possible used the following definition of integrated care by Kodner and Spreeuwenberg (2002) [43] “Integration is a coherent set of methods and models on the funding, administrative, organisational, service delivery and clinical levels designed to create connectivity, alignment and collaboration within and between the cure and care sectors. The goal of these methods and models is to enhance quality of care and quality of life, consumer satisfaction and system efficiency for patients with complex, long-term problems cutting across multiple services, providers and settings. The result of such multi-pronged efforts to promote integration for the benefit of these special patients groups is integrated care.” This definition’s goal of improving the quality of healthcare services is further reflected in Donabedian’s framework on quality of care which uses ‘outcome’ as an approach to assess medical care. This review focuses on patient-related outcomes and according to Donabedian, there are many advantages using patient-related outcomes to assess the quality of care as they tend to be measurable (e.g. length of stay in hospital) and precise (e.g. mortality). However Donabedian’s framework also recognises that other factors than medical care such as technology and time may influence the outcomes assessed [44]. The outcome approach of Donabedian’s framework has been applied to the analysis of the findings of this review in the Discussion section below.

## Methods

### Search strategy

A systematic search of English language literature published between 1^st^ January 1995 and 10^th^ October 2018 in the following 5 electronic databases was undertaken: MEDLINE, EMBASE, Cochrane Library, Web of Science Core Collection and Ageline (EBSCO). Restricting the literature search to 1995 and onwards was based on a bibliometric analysis of literature about integrated care which generated only a small number of studies published before 1995. Key search terms were developed by the research team and included ageing, integrated, care, hospitalisation, admission, readmission, length of stay, satisfaction and mortality. The search terms used are outlined in Appendix A. The protocol has been registered with Prospero (registration number CRD42018110491).

### Eligibility criteria

In this paper, integrated care takes place on either an organisational level e.g. integration between health organisations and social care organisations, or on a systemic level i.e. the full spectrum of services including planning, financing and purchasing is integrated across the entire (or almost entire) health and social care sector [45].

Inclusion and exclusion criteria for considering studies in this review are summarised in Table 1. Studies included were of quantitative, qualitative and mixed-method design undertaken on an organisational or systemic level of integration targeting older people providing objective data on at least one of the following patient-oriented outcomes: mortality; hospital admission; length of hospital stay; hospital readmission; or subjective data on patient satisfaction. For intervention studies, the intervention group had to be compared with a control group receiving standard (non-integrated) care or with baseline measures of the intervention group. The intervention had to consist of pooled recourses (financial or human) on healthcare and social care. Interventions consisting of only multidisciplinary team, care co-ordination and care planning contents were excluded. Intervention studies addressing a single medical disease were also excluded. Due to large contextual differences between health care systems in low-, middle- and high-income countries only studies of integrated care in high income settings, based on the World Bank Groups list of high income countries for 2017, were included in this review. Studies were also excluded if no abstract was available or the study was a review of studies.

**Table 1:** Study inclusion and exclusion criteria.

Population	Older adults

Intervention	Inclusion: Organisational or systemic level of integration; Exclusion: interventions not consisting of pooled resources, interventions for hospitalised patients, interventions focusing on a single medical disease
Comparisons of interest	Comparison of no intervention i.e. usual care or comparison with baseline measures of the intervention group; Exclusion: Studies with no comparison
Outcomes	Any objective measure of hospital admission, length of hospital stay, hospital readmission, mortality, and subjective measure of patient satisfaction
Type of study	Inclusion: studies of any designs published between January 1995 to October 2018 written in English; Exclusion: Studies with no original data e.g. reviews, studies without an abstract

### Study selection and critical appraisal

References retrieved through the systematic searches were managed using Endnote X7 reference management software. Duplicates were removed and the remaining references were independently screened for eligibility by two researchers (FB and AL). Any disagreements were resolved through discussion between reviewers and with a third researcher (JA). Studies meeting the eligibility criteria were assessed for quality using the Critical Appraisal Skills Programme (CASP).

### Data extraction, assessment of risk of bias and synthesis

The data were extracted and organised according to study outcome (patient satisfaction, hospital admission, length of stay, readmission and mortality) and level of health care integration (organisational or systemic) using a standardised data extraction form. The studies included were assessed for risk of biases from six sources (selection, performance, detection, attrition, reporting, conflict of interest) including interwoven risk of bias using relevant checklists for randomised controlled trials, observational studies and qualitative studies developed and provided by the Swedish Agency for Health Technology Assessment and Assessment of Social Services [46,47,48]. The studies were rated low, moderate or high on each of the six sources of bias and the ratings allowed for an interwoven risk of bias for each study. A narrative synthesis enabling handling a wide range of evidence from quantitative and qualitative research was used to compile the results of this review.

## Results

### Results of the literature search

**Figure 1:**
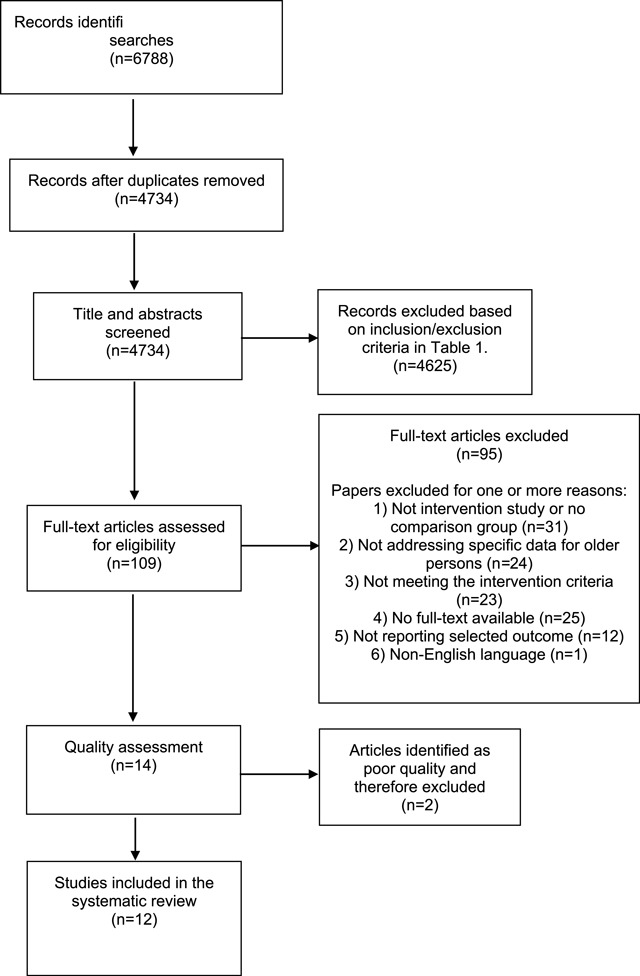
Study selection flowchart.

A flowchart of the literature screening process is outlined in Figure 1. The literature search yielded a total of 6788 studies. After having removed duplicates the remaining 4734 studies were screened for eligibility of which 109 studies were considered potentially eligible and read in full text. Studies not meeting the eligibility criteria (n = 95) were excluded. The remaining 14 articles underwent CASP quality assessment. Results of the quality assessment of 14 studies are shown in Appendix B. Six of the studies were classified as having low risk of bias and five studies had a moderate risk of bias. Two studies were considered to have a high risk of bias and consequently excluded, resulting in a total of 12 studies included in the review.

### Description of the studies identified

The characteristics of the 12 studies included in this review are presented in Table 2. Nine studies were of quasi-experimental design, of which seven had concurrent control group, two studies were randomised controlled trials (RCTs), two were observational studies and one was of qualitative design. Four of the studies evaluated the impact on integrated care on a systemic level and eight studies on an organisational level of integration. Half of the studies were undertaken in North American and the remaining half in Western Europe.

**Table 2:** Characteristics of studies included in this systematic review.

Author, year	Country	Study design	Study subjects	Length of follow-up	Integrated care programme	Outcomes reported

Atherly et al. 2004	USA	survey	n = 402 community-dwellers; n = 235 enrolled to the Program for All-Inclusive Care of the Elderly (PACE) and n = 167 non-PACE community-dwellers	18 months	PACE (The Program for All-Inclusive Care of the Elderly) involves comprehensive integration of medical and social services and care including care planning with family members.	Patient satisfaction
Beland et al. 2006	Canada	RCT	n = 1297 community-dwellers aged >64 years with moderate or severe functional problems (assessed using the Functional Autonomy Measurement System) of which n = 606 assigned to SIPA and n = 624 assigned to the usual services available	22 months	SIPA (System of Integrated Care for Older Persons) a publicly managed and funded system of community-based multidisciplinary teams with full clinical responsibility for delivering integrated care through the provision of community health and social services and the coordination of hospital and nursing home care.	Hospital admission, patient satisfaction, mortality
Bernabei et al. 1998	Italy	RCT	n = 200 community-dwellers aged ≥65 years with frailty based on their physical, mental and cognitive health identified through existing home health services or home assistance programmes; n = 100 intervention and n = 100 controls who received care as usual	12 months	Intervention involving case management and care planning by the community geriatric evaluation unit consisting of 2 case managers (performing assessments, monitoring the provision of services), 1 social worker, 1 geriatrician, nurses and general practitioners.	Hospital admission, length of stay, mortality
Brown et al. 2003	UK	mixed-methods	n = 393 community-dwellers aged >64 years; n = 195 integrated care, n = 198 care as usual	18 months	Joint working primary and social care consisting of two co-located integrated teams, one based in a general practice and the other in a health centre attached to a general practice.	Patient satisfaction
Ham et al. 2003	USA, England	comparative study	Data from medical records presented in numbers per 100 000 population consisting of people aged ≥65 years	n/a	Kaiser-Permanente: a medical care program that involves voluntary enrolment, prepayment for services, comprehensive benefits, preventive medical care, integrated hospital-based health care facilities, and provision of physician services through group medical practice vs NHS (National Health Service): the universal and free healthcare programme in England.	Hospital admission, length of stay
Hebert et al. 2010	Canada	quasi-experimental	n = 920 community-dwellers aged ≥75 years at risk of functional decline (assessed using the Functional Autonomy Measurement System); n = 501 assigned to PRISMA and n = 419 controls receiving care as usual	4 years	PRISMA (Program of Research to Integrate Services for the Maintenance of Autonomy) an embedded model with a single entry point using all the public, private, or voluntary health and social service organisations involved in caring for older people in a given area where every organisation keeps its own structure but agrees to participate under an umbrella system and to adapt its operations and resources to the agreed requirements and processes. Case manager included in PRISMA could be any clinical healthcare professional and is responsible for conducting a thorough assessment of the patient’s needs, planning the required services, arranging patient admission to these services, etc.	Hospital admission, patient satisfaction, length of stay, readmission, mortality
Landi et al. 1999	Italy	quasi-experimental	n = 115 community-dwellers, mean age 77.5 years (+/– 11.7) assessed pre/post intervention	6 months	Case managers and the geriatric evaluation unit designed and implemented individualised care plans in agreement with general practitioners, and determined the services for which patients were eligible. The approved services were then provided by multidisciplinary teams, with the case manager coordinating the delivery and facilitating the integration process between social and healthcare professionals.	Hospital admission, length of stay
Landi et al. 2001	Italy	quasi-experimental	n = 1204 community-dwellers, mean age 77.4 years (+/– 9.7) assessed pre/post intervention	12 months	National model that integrates all the community-based services provided either by the health agency or by the municipality into one “single enter” centre.	Hospital admission, length of stay
Looman et al. 2014	The Netherlands	quasi-experimental	n = 417 community-dwellers aged ≥75 years who were frail (assessed with the Groningen Frailty Indicator); n = 205 assigned to WICM and n = 212 received care as usual	3 months	WICM (Walcheren Integrated Care Model) includes a single entry point system through the general practice known for patient data being shared across teams and focus on prevention. Case managers organise admittance to the required services, the planning and co-ordination of care delivery and periodical evaluation and monitoring of the treatment plan in cooperation with multidisciplinary teams.	Patient satisfaction
Schiotz et al. 2011	USA, Denmark	comparison study	Data from medical records of people aged ≥65 years with one or more of the following 5 chronic conditions: angina, heart failure, COPD, hypertension and diabetes.	n/a	Kaiser-Permanente: a medical care programme that involves voluntary enrolment, prepayment for services, comprehensive benefits, preventive medical care, integrated hospital-based health care facilities, and provision of physician services through group medical practice vs Danish Healthcare system (DHS): the universal and free healthcare programme in Denmark.	Hospital admission, length of stay, readmission, mortality
de Stampa et al. 2014	France	quasi-experimental	n = 428 community-dwellers aged >64 years classified as very frail (assessed using Katz ADL, Lawton IADL, the cognitive performance scale, the depression rating scale, etc.); n = 105 assigned to COPA intervention and n = 323 received care as usual	12 months	COPA (Coordination of care for the elderly) single entry point system connecting primary care and hospital care, home-based geriatric assessment, individualised care plan, interdisciplinary protocols, case manager organises planned hospital visits.	Hospital admission
Tourigny et al. 2004	Canada	quasi-experimental	n = 482 people aged ≥75 years (2/3 living in own home, 1/3 in private seniors’ residence) reporting functional decline (based on Katz ADL, Lawton IADL, etc.); n = 272 in geographical area where ISD is provided and n = 210 in different geographical area where there was no ISD network	5 years	ISD (Integrated service delivery) network of health and social services is a single entry service designed to manage both home and institutional care that involves exchange of clinical information across institutions. The service is run by case managers who develop individual service plans for enrolled patients.	Hospital admission, length of stay, readmission, mortality

### Findings on the outcome measures

**Figure 2:**
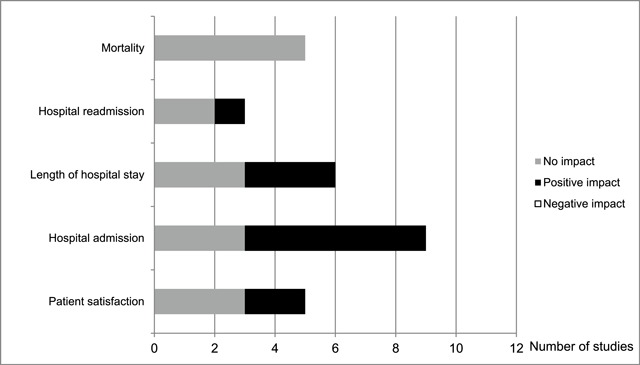
Summary of studies related to each of the patient-related outcomes assessed.

Table 3 presents a summary of the findings for each study. A summary of studies related to each of the patient-related outcomes is presented in Figure 2.

**Table 3:** Summary of findings on patient-related outcomes of integrated care comprising older adults.

Author, year	Level of integration	Patient satisfaction	Hospital admission	Length of stay	Readmission	Mortality

Atherly et al. 2004	Systemic	Significantly greater satisfaction in level of concern and attentiveness displayed by staff (p < 0.01) and patient’s decisions about their care (p < 0.01) but not access to medical specialists (p = 0.54) among PACE-enrolled participants compared to non-participants completing the 23-item PACE satisfaction survey.	n/a	n/a	n/a	n/a
Beland et al. 2006	Systemic	No significant difference between intervention and control groups using the 8-item Client Satisfaction Questionnaire. No effect size reported.	No significant difference between intervention and control groups. No effect size reported.	n/a	n/a	No significant difference between intervention and control groups. No effect size reported.
Bernabei et al. 1998	Organisational	n/a	Significantly reduced risk in the intervention group. HR: 0.74 95% CI 0.56–0.97, p < 0.05	n/a	n/a	No significant difference between intervention and control group. HR 0.99 95% CI 0.89–1.09
Brown et al. 2003	Organisational	No major differences between intervention and control group regarding their satisfaction with the services received – question asked in qualitative interview. No effect size reported.	n/a	n/a	n/a	n/a
Ham et al. 2003	Systemic	n/a	Lower admission rates in KP compared to NHS in 6 of 11 common causes. No effect size reported.	Shorter stay for patients in KP compared to NHS no matter reason for admission. No effect size reported.	n/a	n/a
Hebert et al. 2010	Organisational	Significant improvements in satisfaction with services, delivery of care, and organisation of care and services (all p < 0.001) in intervention group and no changes in control group using the 26-item Health Care Satisfaction Questionnaire	No significant differences in admissions rates in intervention group (rates remained the same) and control group (rates increased) p = 0.578	No difference observed between intervention and control group. No effect size reported.	No difference observed between intervention and control group. No effect size reported.	No difference observed between intervention and control group p > 0.05
Landi et al. 1999	Organisational	n/a	Significant decrease in admission rates post intervention compared to pre intervention p < 0.001	Significantly shorter stays post intervention compared to pre intervention p < 0.01	n/a	n/a
Landi et al. 2001	Organisational	n/a	Significant decrease in admission rates post intervention compared to pre intervention p < 0.001	Significantly shorter stays post intervention compared to pre intervention p = 0.01	n/a	n/a
Looman et al. 2014	Organisational	No significant difference between intervention and control group using study-specific consumer quality index with questions on client-orientation, knowledge of care needs, joint decision making, attention to social-emotional aspects, information, and approach	n/a	n/a	n/a	n/a
Schiotz et al. 2011	Systemic	n/a	Significantly higher admission rates in DHS compared to KP: 5.21 hospitalisations/100 persons 95% CI 5.17–5.26 in DHS and 2.02 hospitalisations/100 persons 95% CI 1.98–2.06 in KP	No significant difference between DHS and KP (4.08 days in DHS, 3.91 days in KP)	Significantly higher readmission rates in DHS compared to KP (OR 1.10 95% CI 1.03–1.16)	No significant difference between DHS and KP (effect size reported for each condition, no effect size on overall findings)
de Stampa et al. 2014	Organisational	n/a	Significantly reduced risk of having at least one unplanned hospitalisation in the intervention group. OR: 0.39 95% CI 0.16–0.98	n/a	n/a	n/a
Tourigny et al. 2004	Organisational	n/a	No significant difference between intervention and control group p = 0.11	No significant difference in average hospitalisation duration between intervention and control group p = 0.28	No significant difference between intervention and control group p = 0.17	No significant difference between intervention and control group p = 0.37

#### Patient satisfaction

Five of the 12 studies included in this review examined how satisfied patients were with the healthcare provided. At 4-year follow-up, Hebert et al. (2010) [49] assessed satisfaction using the 26-item Health Care Satisfaction Questionnaire showing significant improvements in satisfaction with services, delivery of care, and organisation of care in the intervention group compared to the control group. A 24-item survey developed to measure satisfaction in the Program for All-Inclusive Care of the Elderly (PACE) reported patient satisfaction in the form of level of concern and attentiveness displayed by staff, and patient’s decisions about their care, but not in terms of access to medical specialists 18 months after the intervention was introduced [50]. Three studies using different assessment tools (a self-constructed consumer quality index, interview questionnaire and the 8-item Client Satisfaction Questionnaire, respectively) and different length in follow-up (3 months, 18 months and 22 months, respectively) did not report any differences in patient satisfaction with the healthcare provided [51,52,38].

#### Hospital admission

Nine studies reported on hospital admission rates. Two Italian studies observed lower hospital admission rates after the implementation of an individualised integrated home care plan based on a geriatric assessment [53] and after implementation of a “single enter” health and social care centre providing all community-based services available [54], respectively. Similarly, a 12-month intervention study conducted in France observed a significant decline in hospital admission rates [55]. Studies comparing large-scale healthcare programmes showed that the national Danish Healthcare programme, a universal and free of charge care system, has 2.5 times higher rates of hospital admission compared to Kaiser Permanente, a medical programme that involves voluntary enrolment, prepayment for services and provides integrated care [56]. However, when comparing Kaiser-Permanente with the English National Health Service, which is universal and free of charge, similar patterns of hospital admission were reported in both systems [57].

A Canadian intervention study using a 4-year follow-up reported that the number of hospital admissions remained the same in the intervention group but increased in the control group over time albeit not at a statistically significant level [49]. Another two Canadian studies reported no significant differences in hospital admission between the intervention and control groups [58,52].

#### Length of hospital stay

Compared to Kaiser Permanente, the length of stay in hospital was longer in both the Danish Healthcare system [56] and the English National Health Service [57]. The difference observed between the Danish Healthcare system and Kaiser Permanente was however not significant. Some intervention studies reported that compared to the control group, participants in the intervention group stayed fewer number of days in the first 6 months [53] and 12 months [54] after the study was implemented. Contrary, studies with longer follow-up reported no difference between the intervention and control groups after 4 years [49] and 5 years [58], respectively.

#### Patient readmission

Only three of the studies reported on patient readmission to the emergency department after initial hospital admission. Shiotz et al. (2011) [56] reported a small significant difference between Kaiser Permanente and the Danish Healthcare system with higher rates of readmission in Denmark. Tourigney et al. (2004) [58] observed that the number of return visits to the emergency department within 10 days of a first visit was lower in the intervention group compared to the control group but the findings were not statistically significant. Similarly, no significant difference in readmission was observed in the intervention study by Hebert et al. (2010) [49].

#### Mortality

Nearly half of the studies (n = 5) examined mortality as an outcome [58,52,49,56]. None of the studies reported significant changes in mortality rates.

## Discussion

This review has reported on a range of patient-related outcomes assessed in previous studies on integrated care provided to older adults. The findings suggest that integrated care may have a positive impact on hospital admission rates, some positive impact on length of stay and possibly also on patient satisfaction and readmission. Integrated care did not have an impact (positive or negative) on mortality. None of the studies reported integrated care having a negative impact on any of the outcomes assessed.

The positive impact of integrated care on hospital admissions and length of hospital stay is supported by a recent umbrella review of systematic reviews on hospital outcomes in integrated care for the general population [27]. In the umbrella review, 11 of 21 studies reported significantly reduced hospital admissions of which 4 out of 5 intervention studies focused on care of a chronic condition such as heart disease. In our systematic review, apart from the 2 studies that compared hospital admissions in national health programmes [57,56], the remaining 4 intervention studies demonstrating a decline in hospital admissions either involved case management [59,53] or a single entry point system [54,55]. However, no firm conclusions of the impact of case management and single entry point system can be drawn based on these components as 2 other studies in our systematic review using a single entry point system reported no significant reduction in hospital admission rates [58,49]. Besides single entry point systems, case management has previously also shown to be largely ineffective in reducing hospital admission rates [27]. A recent systematic review on complex healthcare interventions targeting older people has concluded that understanding the context including existing routines and the needs of the staff, and having knowledge of the target population are essential to demonstrate effectiveness of the healthcare intervention [60]. Hospital admission rates and length of hospital stay are largely financially driven outcomes [40], and may therefore primarily demonstrate an effect in structural interventions that involve reallocation of funding such as the in Kaiser Permanente [57,56]. Speculatively, time to follow-up may also influence the outcome as studies that showed a reduction in hospital admission rates had a follow-up of 12 months or less [59,53,54,55], whereas studies with a follow-up of 22 months or longer did not report a reduction [58,52,49]. Restricting the eligibility criteria to non-specific conditions may further have influenced this finding as reductions in hospital admissions have been found to be substantially greater in condition-specific admission compared to all-cause admissions [27].

The authors of the recent umbrella review on hospital outcomes in integrated care further reported that length of stay was the outcome most likely to improve with 9 out of 16 reviews reporting positive associations [27]. Nevertheless, the authors reported that gains were often modest, in some studies by as little as 1.5–2 days. Reducing the number of days in acute hospital is essential as prolonged stay increases the risk of hospital-acquired infections and disrupts patient flow due to bed shortages [61]. Thus, in view of the risks of staying in hospital, even a small reduction in length of hospital stay may be beneficial [62]. However, shortening the length of stay to achieve higher patient turnover increases the risk of hospital readmission and may affect the quality of care negatively [63]. In the general population, integrated care models focusing on discharge management for patients seem to be effective in reducing length of hospital stay and hospital readmission [27]. In our review on older adults, it was however impossible to conclude what component(s) of integrated care contributed to reduced hospitalisation due to major differences in the design of the studies. Similarly, we could not draw any conclusions in terms of the potential positive impact on patient satisfaction and readmission because of the inconsistency in the findings observed in the small number of studies reporting on each of these outcomes. For example, five studies reported on patient satisfaction of which 2 reported a positive impact and the remaining 3 studies reported no impact. The inconsistency in the findings on patient satisfaction may be explained by a variety in the patients’ expectations and the health status of the patients as perceived quality of integrated care has previously been shown to be most prominent in older people at the risk of frailty [64]. Many older people regard deteriorating health as normal and may report being satisfied with the care provided because of lower expectations [65]. On the other hand, older people who receive support from informal carers are often very satisfied with their help and may therefore not rank professional social care higher than informal care [50]. This may at least partly explain why older people participating in integrated care interventions are not necessarily more satisfied with the care compared to the control group. However, contrary to these potential reasons, research has also shown that older people, both those who are robust and those who are frail, fear (further) deterioration of health and loss of independence [66]. Hence, inconsistency in the findings on patient satisfaction could also be due to other reasons including data being self-reported and assessed using various questions and scales. For example, the satisfaction survey developed for the PACE study was directed towards community-dwelling older people deemed to be eligible for nursing home care yet still living at home. The applicability of these questions to other patient groups and settings remains however unknown [50].

One of the included studies also showed that increased productivity had a positive impact on patient satisfaction. Despite fewer contacts with nurses, the intervention group reported greater satisfaction with services, the delivery of care and services, and the organisation of care and services, compared to the control group [49]. The authors concluded that their intervention had been successful in targeting those likely to benefit the most and that the findings demonstrated ‘real effectiveness’. Successfully distributing the resources to target those in greatest need is further considered the biggest challenge facing the Swedish health and social care system [12]. Following the patient choice of primary care provider reform introduced in 2010, hundreds of new private primary care practices that operate for profit have been established across the country. Recent research has shown that being able to choose primary care provider has improved access and increased the number of visits to primary care but this has particularly benefitted wealthier people with lower healthcare needs and failed to prioritise those with greater needs [67]. People who consider themselves in need of seeing a medical doctor seem to seek care at the emergency departments rather than primary care clinics where, depending on their health problems, they may be seen by a nurse. Local initiatives have, however, shown that developing and implementing integrated care between hospitals, primary care and social care reduces the number of hospital admissions and readmissions [68,69]. For example, at a primary care clinic in Borgholm, a municipality in the south of Sweden with the oldest population in the country, dramatic changes have been made to improve access to healthcare. This includes patients being guaranteed a same day home visit by a medical doctor, which has reduced the burden on ambulatory staff and the number of visits to the emergency department. Improved exchange of patient information between hospital and primary care following a hospital admission has allowed for quicker follow-up once the patient has been discharged. Such follow-ups include prevention of further illness and thus readmission to the hospital [69]. Another small-scale example of integrated care in Sweden is the Tiohundra project in Norrtälje where the municipality and the county mutually provide all health and social services to the population of Norrtälje. This allows for changes in operations and management to swiftly be translated into changes in the delivery of care. The project involves case managers overlooking the planning and implementation process which is developed together with the patient and their relatives following hospital discharge. The development of strategies for caring of older people with complex needs also involves outlining pathways and plans around transitions in and out of hospital from nursing homes to hospital [68].

The challenge of providing integrated health and social care in a decentralised system where these two facets of care are funded and controlled by different levels of government is not unique for Sweden [68]. It is however clear that the existing fragmented and silo-based health and social care will not be able to meet the complex health and social care needs of the rapidly growing number of older adults with multi-morbidity [70]. Thus, future comprehensive studies using measurable outcomes that allow for statistical assessments of the impact of integrated care are needed to conclude what an optimal health and social care system should involve.

Examining outcomes as measures of quality of care is supported by Donabedian who favours outcomes that are concrete and clearly defined [44]. Donabedian argues that outcomes such as hospital admission rates reflect the influence of health sciences on achievement of certain results. Indeed our findings on hospital admission and length of stay support positive achievements in previous research in health sciences [27]. Nonetheless, Donabedian stresses that the limitations of the outcomes assessed must be recognised including the outcome assessed possibly being irrelevant for the study. When applying Donabedian’s framework to our findings, the complete lack of association between integrated care for older adults and mortality suggests that mortality may not have been a relevant measure. Donabedian also argues that the time aspect can act as a limitation e.g. long periods of time that must elapse before relevant outcomes are manifest, potentially delaying the availability of the results. Thus, mortality may be more relevant when studying younger and middle-aged adults which allow for longer follow-up.

### Strengths and limitations

Strengths of the study include that this is one of the first systematic reviews on non-disease specific integrated care assessing multiple outcomes related to older patients. The search strategy comprised of five different patient-related outcomes of integrated care, was developed together with two well-experienced librarians and applied to 5 different databases. Limitations include that 25 studies were excluded due to full-text versions being unavailable (mainly conference abstracts), and searches did not include grey-zone literature and non-English literature, possibly resulting in relevant data not being taken into account. Further, the number of studies examining each of the outcomes was often small including only 3 eligible studies investigating readmission making it impossible to draw any conclusions. Furthermore, in some of the included studies the authors speculated whether lack of statistical power might have explained the lack of associations [58,49]. Also, due to methodological and interventional heterogeneity of the studies, statistical calculations of objectively assessed outcome measures could not be undertaken. The variety of interventions presented were due to local circumstances, which may not have to be considered a weakness per se. Nevertheless, as a consequence it remains unclear whether the findings are statistically significant.

### Conclusions

This systematic review has explored multiple patient-related outcomes of integrated care targeting older people. The findings suggest that integrated care may have a positive impact on hospital admission rates in older age. Integrated care may also positively influence the length of hospital stay and possibly also patient satisfaction and readmission. In contrast, integrated care did not have an impact on mortality. However due to lack of robust findings, the effectiveness of integrated care on patient-related outcomes in later life remain largely unknown. Whilst this review has tried to identify patient-related outcomes important in integrated care provided to older adults, further theory-based research is needed to assess the effect on these outcomes in integrated care settings.

## Appendix A

Search terms used

Population

aged

aging/ageing

old/older

elderly/elders

geriatric*

psychogeriatric*

dementia/demented

alzheimer*

community-dwelling

late-life*

retired/retirement

senior citizen*

pensioner*

Integrated care

Delivery of Health Care

Integrat*

Continuity of Patient Care/

Comprehensive Health Care/

Coordinated/co-ordinated

Comprehensive

Collaborative

Transmural

Continuity

interdisciplinar*

multidisciplinar*

multiprofessional

multiagency

multisector

seamless

shared

whole

interface

locality

interaction

modelling

health*

care*

delivery

system/systems

service*

clinic/clinics

care or disease or health

management/managed

community

program*

plan*

team*

Patient-related outcomes

surviv*

mortality

hospitalization

Length of Stay

readmission*/re-admission*

Patient Admission

Patient Readmission

satisfact*

Patient Satisfaction

## Appendix B

Results of quality assessment of the included quantitative studies

**Table:**

Author (year)	Study design	Selection bias	Risk of performance bias	Risk of detection bias	Risk of attrition bias	Risk of reporting bias	Risk of conflict of interest	Interweaved risk of bias

Atherly et al. (2004)	Quasi-experimental	Moderate	Low	Moderate	Moderate	Low	Moderate	Moderate
Beland et al. (2006)	RCT	Low	Low	Low	Moderate	Low	Low	Low
Bernabei et al. (1998)	RCT	Low	Low	Low	Low	Low	Low	Low
Ham et al. (2003)	Retrospective cohort	Moderate	Moderate	Low	Low	Low	Low	Low
Hebert et al. (2009)	Quasi-experimental Study	Moderate	Moderate	Moderate	Low	Moderate	Low	Moderate
Landi et al. (1999)	Quasi-experimental study	Low	Low	Low	Low	Moderate	Moderate	Low
Landi et al. (2001)	Quasi-experimental study	Low	Low	Low	Moderate	Low	Moderate	Low
Looman et al. (2014)	Quasi-experimental Study	Low	Low	Moderate	Low	Low	Moderate	Low
Schiotz et al. (2011)	Retrospective cohort	Moderate	Moderate	Moderate	Low	Low	Low	Moderate
de Stampa et al. (2014)	Quasi-experimental Study	Low	Low	Low	Moderate	Low	Low	Low
Tourigny et al. (2004)	Quasi-experimental	Low	Low	Low	Low	Low	Moderate	Low

Results of the quality assessment for the qualitative study

**Table:**

Author (year)	Study design	Well defined aim	Risk of Selection bias	Risk of data collection bias	Risk of analysis bias	Risk of reporting bias	Interweaved risk of bias	

Brown et al. (2003)	Non-randomised comparative study	Yes	Low	Low	Low	Low	Low	
